# Activity in Occipito-Temporal Cortex Is Involved in Tool-Use Planning and Contributes to Tool-Related Semantic Neural Representations

**DOI:** 10.1162/nol_a_00159

**Published:** 2024-12-03

**Authors:** Simon Thibault, Eric Koun, Romeo Salemme, Alice C. Roy, Véronique Boulenger, Claudio Brozzoli

**Affiliations:** Integrative Multisensory Perception Action & Cognition Team (ImpAct), Centre de Recherche en Neurosciences de Lyon, INSERM U1028, CNRS UMR5292, Lyon, France; University of Lyon, Lyon, France; Laboratoire Dynamique du Langage, CNRS UMR5596, Lyon, France; Aging Research Center (ARC), Department of Neurobiology, Care Sciences and Society, Karolinska Institutet, Stockholm, Sweden

**Keywords:** embodied cognition, fMRI, language, semantics, tool use

## Abstract

Tool use and language are highly refined human abilities which may show neural commonalities due to their potential reciprocal interaction during evolution. Recent work provided evidence for shared neural resources between tool use and syntax. However, whether activity within the tool-use network also contributes to semantic neural representations of tool nouns remains untested. To this aim, we identified the tool-use planning network with functional magnetic resonance imaging while participants used pliers. The very same participants underwent a semantic priming task including two categories, tool nouns and animal nouns, to highlight the respective underlying networks. With multivariate analyses of the activation neural patterns, we tested whether activity in tool-use brain clusters takes part in the neural representation of tool nouns as compared with animal nouns. The results revealed that word semantic categories were decoded within the left occipito-temporal cortex activated by preparing to use a tool, with similar patterns of brain activity for words within the same category. In addition, in the same area, neural activations for tool nouns were found to be higher than those for animal nouns. These findings suggest that activity in tool-use related brain areas encodes semantic information separately for tool nouns and animal nouns, thus supporting the embodiment of tool-noun processing in the tool-use sensorimotor network.

## INTRODUCTION

Tool use and language are advanced human abilities which might have co-evolved and influenced one another during evolution (Ponce de León et al., 2021). This potential co-evolution predicts that tool use and linguistic processes may rely on common neural and cognitive resources (Stout & Chaminade, 2012). Recently, it has been evidenced that tool use and syntax share cognitive processes and neural substrates within the basal ganglia (BG; Thibault et al., 2021). The fundamental question therefore is whether tool use also shares other crucial components with language. Tool use relies on a left fronto-parietal network, encompassing the inferior frontal gyrus (IFG) and inferior parietal lobule (IPL; Brandi et al., 2014; Peeters et al., 2009), as well as subcortical structures like the BG (Choi et al., 2001; Hermsdörfer et al., 2007). The left fronto-parietal stream supports planning and execution of tool-use movements (Brandi et al., 2014), while the BG may handle the sequential and hierarchical aspects of tool use (Choi et al., 2001; Thibault et al., 2021). Furthermore, activity in the occipito-temporal cortex (OTC) contributes to the visual recognition of tools (Chao et al., 1999; Schone et al., 2021), as well as to the observation (Peeters et al., 2009) and actual execution of tool-use actions (Brandi et al., 2014).

Remarkably, the line of research investigating the neural bases of language has shown that the aforementioned brain areas are also critical for processing the semantic content of action-related words. Verbs or sentences depicting actions, tool nouns, or object nouns recruit the left IFG (Aziz-Zadeh et al., 2006; Boulenger et al., 2009; Carota et al., 2017; Cattaneo et al., 2010; Hauk et al., 2004) as well as the left IPL (Aflalo et al., 2020; Boronat et al., 2005; Chao & Martin, 2000). Moreover, reading object nouns or viewing and naming their pictures were shown to involve the left OTC (Carota et al., 2017; Chao et al., 1999; Martin & Chao, 2001; Wurm & Caramazza, 2019). Encoding of tool-related semantic representations could therefore rely on the sensorimotor network involved by refined sensorimotor skills such as tool use.

Is the recruitment of similar brain areas for tool use and semantic processing of tool nouns more than anecdotal, therefore reflecting shared neurocognitive resources? In the framework of the embodied cognition theory (Allport, 1985; Barsalou, 1999, 2008; Barsalou et al., 2003; Borghi & Barsalou, 2021; Buccino et al., 2016; García & Ibáñez, 2016; Pulvermüller, 2005; Pulvermüller & Fadiga, 2010), previous research has pointed to a role of sensorimotor areas in semantic representations (Aflalo et al., 2020; Aziz-Zadeh et al., 2006; Hauk et al., 2004; Kemmerer et al., 2012). In its strong version, this theory predicts that there is no central module for cognition, and that high-level cognitive functions such as language are in part grounded in sensorimotor processes (Pezzulo et al., 2013). For instance, semantic content evoking manual actions (e.g., to grasp, to throw) has been found to elicit brain activity in areas responsible for hand sensorimotor control (Aziz-Zadeh et al., 2006; Boulenger et al., 2009; Desai et al., 2010; Hauk et al., 2004; Tettamanti et al., 2005). However, most of this work often did not concomitantly assess the brain network underlying the actual execution of actions. When it comes to nouns referring to manipulable objects, some evidence also suggests an involvement of the sensorimotor network extending beyond primary sensorimotor areas and including the IFG, IPL, and OTC that are also activated by tool use (Carota et al., 2017; Chao et al., 1999; Chao & Martin, 2000; Martin & Chao, 2001; Perani et al., 1999). However, so far, a co-localization of sensorimotor and linguistic computations has mostly been assumed from motor and linguistic neuroimaging data acquired in distinct samples of participants (see Ishibashi et al., 2016, for a meta-analysis evaluating tool-related cognition). Furthermore, these studies tested the network recruited by the observation of tool pictures (Chao et al., 1999; Chao & Martin, 2000; Perani et al., 1999) or of tool-use actions (Aflalo et al., 2020; Wurm & Caramazza, 2019) rather than by the real execution of these actions, which may entail at least partly different networks (see Courson & Tremblay, 2020). Therefore, at best, such previous work allows for a conclusion that reading tool nouns recruit the same network as observing pictures of tools (Perani et al., 1999). In line with this interpretation, a recent meta-analysis revealed that action language (e.g., action verbs) shows a closer correspondence to action observation than to action production (i.e., execution or imitation; Courson & Tremblay, 2020). Whether this relation is held with specific action-related language such as tool nouns and specific actions such as actual tool use remains unclear. Thus far, most studies that investigated the contribution of the sensorimotor circuits to language, action verbs specifically (e.g., Garofalo et al., 2022), focused on the primary sensorimotor areas (Gough et al., 2012; Klepp et al., 2015, 2019; Visani, Garofalo, et al., 2022; Visani, Sebastiano, et al., 2022). However, tool use is known to extend this network by also recruiting the IPL, IFG, and OTC (Brandi et al., 2014; Chen et al., 2023; Peeters et al., 2009), suggesting that these regions may also participate in tool noun processing. As a matter of fact, imaging studies on the functional relationship between the processing of tool nouns and real tool use are crucially lacking. The present study aimed to fill this gap by testing whether tool nouns are represented within the network subserving the actual use of tools. To do so, we employed a lexical decision task and compared the neural activities elicited by tool nouns and animal nouns. This latter category was selected given the clear contrast it offers with tool nouns, as it refers to animated entities and it does not evoke any specific manipulation or functional use as tools do. The crucial hypothesis that co-localization reflects shared processes between tool use and semantic processing of tool nouns indeed remains untested. To this aim, one would rather test in the same sample of participants, whether the activity in tool-use related areas contributes to semantic processing of tool-related contents compared to animal-related contents. To tackle this issue, we assessed both functions (i.e., semantic processing of tool nouns and tool use) in the exact same group of participants. Through multivariate neural pattern analyses, we tested the hypothesis that activity in specific nodes of the tool-use network (i.e., ensemble of brain regions involved in tool use, including planning and/or execution, as opposed to non-use behavior associated with tools, like observation of tool pictures), allows to decode the word semantic categories, with more similar neural representations for words within the same semantic category. This would be proof that semantic categories (here, tool nouns vs. animal nouns) can be distinguished based on their neural responses within the tool-use-related areas, and that these areas contribute to semantic processing and more specifically to neural representations of tool nouns.

## MATERIALS AND METHODS

### Participants

FMRI acquisition included 24 (11 females and 13 males) right-handed, French native participants. All had normal or corrected-to-normal vision, and they reported no history of neurological, auditory, language, or sensorimotor deficits. Four participants were excluded; two did not fulfill the familiarization performance requirements before any neuroimaging acquisition, one dropped out after the inclusion phase, and one was removed from analyses due to substantial head movements (several runs with movements above 1.5 mm). Hence, we analyzed the data of 20 participants with the following sociodemographic characteristics and manual preference: 10 females and 10 males; mean age ± *SD*: 24 ± 4 yr old; higher education level: 3 ± 2 yr; mean score on the Edinburgh handedness inventory (Oldfield, 1971): 0.93 ± 0.09. The protocol conformed to the Helsinki declaration and was approved by a national ethical committee (46/17, OUEST IV). All participants gave their written consent prior to the study and received compensation of 110 euros.

### Tasks

#### Tool-use task

**Figure 1.**
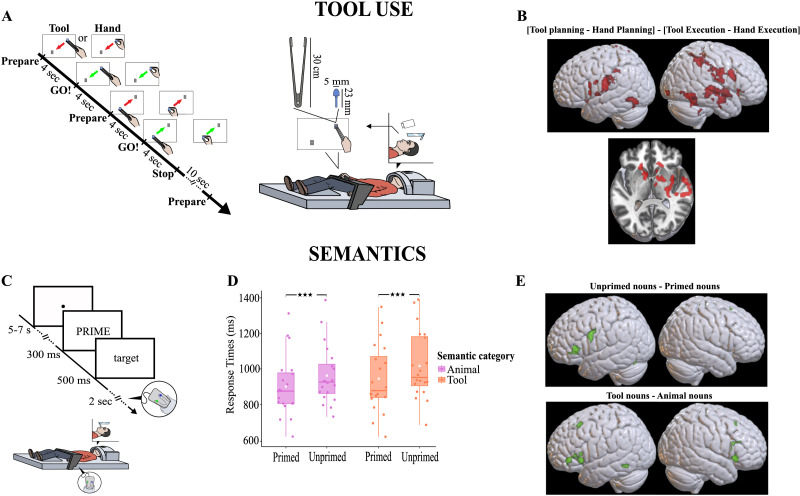
Brain networks for tool use and semantics. (A) Set-up and timing of the events for the motor task in the scanner. (B) Brain activations for tool-use compared to free-hand planning derived from the contrast [Tool planning − Hand planning] − [Tool execution − Hand execution], uncorrected maps at *p* < 0.001 (see Tables S1–S2 for clusters passing the family-wise error (FWE) correction at the cluster level). (C) Set-up and timing of the events for the semantic priming task in the scanner. (D) Semantic priming effect. Irrespective of word category, responses were faster for semantically primed than for unprimed targets (main effect of Priming [*χ*^2^_(1)_ = 25.78, *p* < 0.001]). Tool nouns were processed more slowly than animal nouns (main effect of Semantic category [*χ*^2^_(1)_ = 14.35, *p* < 0.001]). The response accuracy only revealed a trend for the effect of Semantic category: Participants tended to be less accurate for tool nouns than for animal nouns (Tool nouns = 96.3 ± 0.9 % vs. Animal nouns = 99.1 ± 0.4 %, [*χ*^2^_(1)_ = 3.79, *p* = 0.051]). (E) Brain activations for semantic priming derived from the contrast Unprimed nouns − Primed nouns for the top panel and Tool nouns − Animal nouns for the bottom panel, uncorrected maps at *p* < 0.001 (see Tables S1–S2 for clusters passing the FWE correction at the cluster level).

The participants were asked to use a pair of 30-cm-long pliers with their right hand in order to move a peg on a plastic board (Quercetti, Torino, Italy) between a starting point and an end point, materialized by visual landmarks and separated by 9 cm (Figure 1A). The participants were lying in a resting position and were instructed to wait for a pure tone signal delivered through a magnetic resonance imaging (MRI) compatible device (Optoacoustics OptoACTIVE-two-way noise cancellation communication system, Mazor, Israel). A single pure tone required the participant to prepare the movement, while a double tone 4 s later indicated the Go for the action. In order to perform the requested movements, the described sequence was repeated twice: grasping the peg to displace it from the start to the end point, and then grasping it again to move it back to its initial position. The whole sequence (4 s planning – 4 s execution – 4 s planning – 4 s execution – 10 s rest) was repeated 15 times in a single run. The participants had to press a button with their left index finger if a peg fell (i.e., to indicate the missed sequence) and then grab a new peg from the left side of the plastic board. The few missed trials (lower than 0.5%) were modeled separately. As a control, in a distinct run, the same task was performed with the free hand. This allowed us to define the contrast aimed at identifying the tool-specific neural network. The motor task device was placed at a reachable distance in front of the participants and made visible with a double mirror mounted onto the head coil. To minimize elbow and shoulder movements, the participants’ right upper arm was strapped to the trunk. The scripts controlling the audio sequence of instructions in the scanner were delivered with Presentation software (Neurobehavioralsystems, 2024).

#### Semantic priming task

A semantic priming paradigm with a lexical decision task allowed to assess semantic processing. In such a paradigm, prime-target word pairs that are semantically related or not are presented and participants have to make a lexical decision on the target. Stimuli used as primes and targets were French words belonging to two distinct semantic categories: animal nouns and tool nouns. Pseudowords were created by changing two letters from tool and animal nouns while following the French phonotactic rules (i.e., words remained pronounceable and orthographically legal). Pseudowords were only presented as targets, never as primes. The choice of a semantic priming task aimed to specifically highlight the neural correlates underlying word semantic processing. Behaviorally, semantic priming occurs as a facilitation of the response to targets when they are semantically related to primes (Kotz et al., 2002; Matsumoto et al., 2005). At the neural level, this is characterized by a repetition suppression effect, namely reduced brain activation when two semantically related words are presented (i.e., primed condition; Kotz et al., 2002; Matsumoto et al., 2005). By contrast, when the prime and target within a pair are unrelated (i.e., unprimed condition), the neural signal increases upon target presentation (Grisoni et al., 2016; Lau et al., 2013). Hence, the comparison of the neural activity evoked in the unprimed and primed conditions informs about the brain areas reliably activated by target words and their underlying semantic category (Kotz et al., 2002; Matsumoto et al., 2005).

For our task, the selection of word stimuli was based on an anonymized online survey. A total of 574 French native participants (mean age ± *SD* = 23.92 ± 4.39) judged the imageability and manipulability of a subset of a larger sample of 374 object nouns, on a seven-point Likert scale. They could also report whether a word was unknown to them. An additional 246 participants (mean age ± *SD* = 26.59 ± 6.01) judged the imageability of a subset of 220 animal nouns. Because the participants received only a subset of words (i.e., 52 for object nouns and 74 for animal nouns) and not the full list, each word was quoted 82 times (i.e., by 82 participants). Overall, 147 object nouns with manipulability and imageability scores equal or above five (out of seven) were selected as tool nouns. Furthermore, 172 animal nouns with an imageability score equal or above five were retained. Words from the two semantic categories were selected to obtain two sets of 70 tool nouns and 70 animal nouns matched for psycholinguistic variables (written and oral frequencies, numbers of syllables, letters and orthographic neighbors) as verified with the Lexique 3.80 database (New et al., 2004; see Table S3 in the Supporting Information, available at https://doi.org/10.1162/nol_a_00159). The two sets of animal and tool nouns were then each divided into five lists of 14 words each. The resulting 10 lists (5 for animal nouns and 5 for tool nouns) were matched for the same aforementioned psycholinguistic variables (Table S3). Each list was uniquely assigned to a function in the priming paradigm (either prime or target) so as to create five experimental target conditions: Tool Primed, Tool Unprimed, Animal Primed, Animal Unprimed, and Pseudowords.

For words, primes and targets were associated by pairs in four conditions: a prime could be followed by a target either from the same semantic category (i.e., Tool Primed and Animal Primed) or from a different category (i.e., Tool Unprimed and Animal Unprimed). In case of primed conditions, the words within the pair were always different. In a fifth condition, the target was a Pseudoword, following either an Animal or a Tool prime. The prime–target pairs were the same for all participants.

During the experiment, following a fixation dot displayed for 500 ms, a prime word was visually presented in uppercase for 300 ms and immediately followed by a target word in lowercase for 500 ms (Figure 1B). Participants were instructed to indicate as quickly and correctly as possible whether the target was a word or a pseudoword by pressing one of two buttons with their left index or middle finger. They had a maximum of 2 s after target onset to respond. The button-response association was counterbalanced across participants. The intertrial period was jittered between 5 and 7 s.

Overall, participants underwent 84 trials across the five experimental conditions: 14 prime–target pairs were presented for each of the four-word conditions and 28 prime–target pairs for pseudowords (Table S4). The stimuli were visible through the mirror, and the task script was programmed onto Psychtoolbox (Version 3; Kleiner et al., 2007) running on MATLAB (MathWorks, n.d.).

### Procedure

The experiment consisted of an inclusion session to familiarize participants with the tasks as well as to ensure that the individual level of performance met the inclusion criteria. Short versions of the semantic and motor tasks were proposed. The requirements to perform the functional MRI (fMRI) session were at least six correct responses in the lexical decision task (over 9 trials including 6 words and 3 pseudowords). This procedure aimed to maximize the chances of collecting a sufficient number of correct and analyzable trials during the neuroimaging acquisition. The motor task for inclusion consisted of performing two blocks with the tool and two blocks with the free hand. In each block, the participants performed the task on the Grooved Pegboard test and were instructed to insert 10 pegs as quickly as possible on the two first rows. To be included in the experiment, they were required to insert the 10 pegs in less than 5 min on average for the two tool-use blocks and in less than 1 min on average for the two free-hand blocks. The participants took part in two different fMRI sessions separated by two days. For each session, the participants performed an anatomical acquisition (T1-weighted), followed by motor (tool-use and free-hand) and linguistic runs in a counterbalanced order. The semantic task was conducted in a session that also included a phonological task, while two additional tasks were proposed in another session: one assessing syntactic processing and the other, as a control, assessed working memory. The session order was counterbalanced between participants. For the sake of clarity this manuscript reports only on the results of the semantic task.

Functional and anatomical MRIs were acquired with a Siemens Prisma 3T scanner (Siemens Medical Systems, Erlangen, Germany) with a gradient echo EPI sequence, with TE = 30 ms and TR = 2,400 ms. Volumes were acquired with 44 interleaved slices of 3.3 mm thickness (3 × 3 × 3.3 mm voxel size) aligned to the AC-PC plane. Overall, 171 volumes were acquired for each motor block and 303 for the semantic task. T1-weighted images were acquired with a 1-mm isotropic voxel and a generalized autocalibrating partial parallel acquisition (GRAPPA) acceleration factor of 2 (TE = 3.8 ms, TR = 3,000 ms).

### Analyses and Statistics

#### Behavioral analyses

For the semantic task, response times (RTs; i.e., time interval from the display of the target word to the participant’s response) and response accuracy were measured to index semantic performance. Statistics on these data were run in RStudio with the afex package (Singmann et al., 2020). A linear mixed model (LMM; Baayen et al., 2008) was used on RTs and included Semantic Category (Tool nouns vs. Animal nouns) and Priming (Unprimed vs. Primed) as fixed-effect within-subject factors. In theory, models including all the random effects (supported by the design) would lead to the most informative solution. However, this may result in convergence issues while a negligible amount of information is added to the model, or in overfitting (Bates et al., 2015). To address this issue, random effects were added sequentially and their effects on the model fit were assessed with likelihood ratio tests (LRT). Residuals of each model were tested and the one with the significantly lower deviance assessed by a chi-squared (*χ*^2^) test was chosen (see Blini et al., 2018, for a similar procedure). Concretely, we started with only Subjects as random intercept and added Semantic Category and then Priming as random slopes (and their interaction). This was tested sequentially, meaning that each random effect was kept in the model as long as the LRT supported its contribution with respect to the previous model using a simpler random-effects matrix. For our LMM on RTs, the random slopes for Semantic Category and Priming were included (but not their interaction as the LRT was not significantly different) and, with Subjects as random intercept. A generalized linear mixed model (GLMM) testing a binomial distribution was performed on response accuracy. Both Semantic Category and Priming were used as fixed-effect within-subject factors. Using the same sequential strategy described above, only Semantic Category was considered as random slope (adding Priming as random slope did not significantly change the LRT) and Subjects as random intercept. The significance of the fixed effects was tested with an LRT, which follows a *χ*^2^ distribution, meaning *χ*^2^ is the statistical parameter reported and used for estimating each factor significance (*p* < 0.05). All the results are reported as the mean ± *SEM*.

#### fMRI preprocessing

For preprocessing of the fMRI data, we used fMRIPrep (Version 20.2.0; fMRIPrep, 2016), a pipeline aiming to conduct robust and reproducible preprocessing of fMRI data (Esteban et al., 2019). The fMRIPrep pipeline generates a file describing the preprocessing procedure applied (available in the Supporting Information). In short, head motion parameters were estimated, then the images were corrected for magnetic field inhomogeneity from the fieldmap images. Following these steps, the images were slice-timing corrected and co-registered to the T1w image, first in native space and then in the MNI space. In contrast to other software, fMRIPrep does not apply any spatial smoothing of the data.

#### fMRI univariate analyses

The Statistical Parametric Map 12 (SPM12; Ashburner et al., 2021) was used for the univariate analyses. Data were smoothed with a Gaussian kernel of 8 × 8 × 8 mm. At the first level, each participant’s hemodynamic responses were modeled by the convolution of the canonical hemodynamic response with a box-car function. Each motor block was designed with planning, execution, and rest, as well as missed trials and head movements. Both directions of the movements (back and forth) were taken into consideration together. For the semantic priming task, we modeled the prime–target association (i.e., 0.8 s from the prime onset) separately for Tool Primed, Tool Unprimed, Animal Primed, Animal Unprimed, and Pseudowords. The head movements, incorrect trials as well as the remaining time allotted to give the response (i.e., 1.5 s) were modeled in separate regressors.

At the second level, we conducted within-subjects analyses of variance (ANOVAs) to identify the general network underlying the motor task. To do so, we entered the parameter estimates (i.e., beta) against baseline obtained for each condition of interest and each subject. We computed the interaction contrast highlighting the specific tool-use planning neural network with respect to free-hand planning and the overall tool-use execution network. This contrast allowed us to identify the voxels specifically activated for tool-use planning above and over the three other motor conditions (tool-use execution, free-hand execution, and free-hand planning; see Garcea & Buxbaum, 2019, for a similar approach):

$$\text{Tool}-\text{use}\;\text{planning}\;\text{network}=\left[\left(\text{Tool}-{\text{use}}_{\text{planning}}-\text{Free}-{\text{hand}}_{\text{planning}}\right)-\left(\text{Tool}-{\text{use}}_{\text{execution}}-\text{Free}-{\text{hand}}_{\text{execution}}\right)\right].$$

We employed the exact same approach to test for the specific tool-use execution network with the following contrast:

$$\text{Tool}-\text{use}\;\text{execution}\;\text{network}=\left[\left(\text{Tool}-{\text{use}}_{\text{execution}}-\text{Free}-{\text{hand}}_{\text{execution}}\right)-\left(\text{Tool}-{\text{use}}_{\text{planning}}-\text{Free}-{\text{hand}}_{\text{planning}}\right)\right].$$

For the semantic task, we conducted within-subjects ANOVAs to identify the general semantic neural network. As for the motor task, we entered the parameter estimates against baseline obtained for each condition of interest and each subject. To identify the main effect of semantic priming, given that pairs in the primed conditions may induce a repetition suppression effect (Kotz et al., 2002; Matsumoto et al., 2005), we considered the voxels more activated for unprimed in comparison to primed nouns:

$$\text{Semantic}\;\text{priming}\;\text{network}=\left[\text{Unprimed}\;\text{nouns}-\text{Primed}\;\text{nouns}\right]$$

Then we studied the main effect of semantic category by considering separately tool nouns and animal nouns, to evidence the network supporting each category irrespective of the priming manipulation:

$$\text{Semantic}\;\text{tool}\;\text{category}\;\text{network}=\left[\text{Tool}\;\text{nouns}-\text{Animal}\;\text{nouns}\right]$$

and

$$\text{Semantic}\;\text{animal}\;\text{category}\;\text{network}=\left[\text{Animal}\;\text{nouns}-\text{Tool}\;\text{nouns}\right]$$

For the sake of consistency with the general literature on semantics, we also investigated the repetition enhancement and the lexical decision networks:

$$\text{Semantic}\;\text{repetition}\;\text{enhancement}\;\text{network}=\left[\text{Primed}\;\text{nouns}-\text{Unprimed}\;\text{nouns}\right]$$

and

$$\text{Lexical}\;\text{decision}\;\text{network}=\left[\text{Words}-\text{Pseudowords}\right]$$

To guarantee the reliability of the results, for each analysis, we reported each cluster at the whole brain level, containing more than 10 contiguous voxels, with a *p* value below the 0.001 threshold uncorrected for multiple comparisons. Furthermore, the motor contrast was submitted to an exclusive mask defined at 0.05 uncorrected for multiple comparisons, with the aim of ruling out the contribution of the interaction’s second component (i.e., for Tool-use planning network, the contribution Free-hand execution > Tool-use execution was masked). Clusters passing the family-wise error (FWE) correction for multiple comparisons at the cluster level are highlighted in Table S1 and Table S2.

#### fMRI multivariate neural pattern analyses

We selected regions of interest (ROIs) from the tool-use planning neural activity (see Results). Note that as the tool-use execution neural activity was confined to visual areas (i.e., outside the semantic network), no crucial ROI was identified and used for subsequent analyses. For ROIs selected from tool-use planning, we saved a mask of all the significant voxels (*p* < 0.001 uncorrected) for clusters of interest. These masks were derived from the group (i.e., second level) brain map and subsequently applied to each participant. Overall, we retained three ROIs of the tool-use planning network also known for their involvement in semantics. The three tool-use clusters were the left IPL (size = 150 voxels), left OTC (size = 76 voxels), and left IFG (size = 28 voxels). The ROIs localization was controlled with the Automated Anatomical Labelling (AAL) Atlas (Tzourio-Mazoyer et al., 2002). The IPL mostly encompassed voxels in the inferior portion of the postcentral gyrus (71%) and the anterior part of the supramaginal gyrus (29%). The OTC mostly encompassed voxels within the inferior occipital gyrus (50%) and the posterior portions of the inferior (26%) and middle (17%) temporal gyri, the remaining 7% encompassing surrounding areas. Finally, the IFG included voxels of the precentral gyrus (20%) and the pars opercularis of the IFG (80%). Despite the IFG mask being small and the cluster not reaching significance after correction for multiple comparisons (see Table S1), we included this mask in our analyses given our a priori hypothesis on the contribution of this area to both tool use and semantic processing (see Introduction). Using a more lenient cluster-forming threshold of *p* = 0.005, this IFG cluster was much larger (size = 924 voxels) and covered a very large range of frontal lobe regions. Using such a large cluster is, however, unlikely to be informative for our purpose since the semantic signal may substantially differ within the entire ROI. Given the lesser statistical robustness of the IFG cluster, the analysis performed in this region will be considered exploratory. For the semantic task, first-level analyses were run on non-smoothed data and included the same regressors as for the univariate analyses. We used the CoSMoMVPA toolbox (Oosterhof et al., 2016) to extract, for each participant, the *t* value at each voxel within a given ROI. For the multivariate analyses, we calculated the similarity of the neural patterns using the cosine similarity across a set of conditions. We considered four conditions of interest (i.e., Tool Primed, Tool Unprimed, Animal Primed, Animal Unprimed). Overall, we calculated for each subject the cosine similarity for each of the six pairs of conditions: Tool Primed/Tool Unprimed; Animal Primed/Animal Unprimed; Tool Primed/Animal Primed; Animal Unprimed/Tool Unprimed; Tool Primed/Animal Unprimed; Animal Primed/Tool Unprimed. To test whether the tool-use network decodes tool nouns from animal nouns, we reasoned that target words from the same semantic category (i.e., Tool or Animal nouns) would elicit more similar neural patterns (i.e., within-category similarity) than target words from different semantic categories (across-category similarity). Hence, we tested whether the neural pattern similarities of Tool Primed/Tool Unprimed and Animal Primed/Animal Unprimed would be greater than the similarities of all the other pairs of conditions, where the target words belong to different semantic categories. For each subject and within each ROI, we calculated the following contrast where the similarity for each pair was measured with the cosine similarity:

(1)  [(Tool Primed/Tool Unprimed) + (Animal Primed/Animal Unprimed)]/2 − [(Tool Primed/Animal Primed) + (Animal Unprimed/Tool Unprimed) + (Tool Primed/Animal Unprimed) + (Animal Primed/Tool Unprimed)]/4

In addition, we also tested two further contrasts assuming differences between categories within an ROI, namely whether one semantic category could be decoded with a stronger within-category similarity than the other category (and than across-category pairs). The two contrasts assessed a stronger within-category similarity for, respectively, tool nouns and, as a control, animal nouns:

(2)  (Tool Primed/Tool Unprimed) − [(Tool Primed/Animal Primed) + (Animal Unprimed/Tool Unprimed) + (Tool Primed/Animal Unprimed) + (Animal Primed/Tool Unprimed) + (Animal Primed/Animal Unprimed)]/5

and

(3)  (Animal Primed/Animal Unprimed) − [(Tool Primed/Animal Primed) + (Animal Unprimed/Tool Unprimed) + (Tool Primed/Animal Unprimed) + (Tool Primed/Tool Unprimed)]/5

To assess whether this difference in similarity differed from zero within a given ROI (i.e., greater within- than across-semantic-category similarity), we performed 10,000 permutations by randomly flipping the sign of the participants’ scores in order to obtain a null distribution. The probability for observing a significant effect under the null hypothesis was thresholded at 0.05 right-tailed (i.e., a score above zero is expected) and calculated from the proportion of values of the null distribution superior to the observed score. Thus, if this proportion is smaller than 0.05, the observed score is considered significant and different from the chance level set at zero. We applied a false rate discovery (FDR) correction on this probability to correct for multiple comparisons. Importantly, the multivariate contrast (1) tested whether both word semantic categories can be decoded within an ROI. In case this contrast was significant for one ROI, to test the prediction that tool nouns specifically recruit the tool-use network, we ran a follow-up analysis, where we averaged the neural signal across all the voxels for each subject and each semantic condition separately (i.e., Tool Unprimed, Tool Primed, Animal Unprimed and Animal Primed nouns). We then performed an ANOVA with Semantic Category (Tool vs. Animal) and Priming (Unprimed vs. Primed) as within-subject factors. This analysis aimed to identify which semantic condition (i.e., Tool Unprimed, Tool Primed, Animal Unprimed and Animal Primed) induced the strongest neural activity within the tool-use network. Post hoc tests were corrected for multiple comparisons with FDR correction.

## RESULTS

### Tool-Use and Semantic Neural Networks

Planning an action with the tool, rather than with the hand, recruited a network encompassing bilaterally the BG, the OTC, and the supramarginal portion of the IPL (*p* < 0.05 FWE cluster corrected, *p* < 0.001 cluster-forming threshold; Figure 1B and Table S1A), in addition to the bilateral ventral premotor part of the IFG (*p* < 0.05 FWE cluster corrected on the right and *p* < 0.001 uncorrected on the left hemisphere). By contrast, executing an action with a tool, compared to the hand, recruited the secondary visual area bilaterally (*p* < 0.05 FWE cluster corrected, *p* < 0.001 cluster-forming threshold; Table S1B). In the semantic task, behavioral results revealed classical priming effects: Participants responded faster to semantically primed as compared to unprimed words, irrespective of their category (*χ*^2^_(1)_ = 25.78, *p* < 0.001; Figure 1D). Furthermore, responses were slower (*χ*^2^_(1)_ = 14.35, *p* < 0.001), and tended to be less accurate (*χ*^2^_(1)_ = 3.79, *p* = 0.051), for tool than for animal nouns. No interaction between Priming and Semantic category was found for RTs or accuracy (*χ*^2^_s_ < 0.59, *p*_s_ > 0.44; Tool Primed = 945 ± 4 3 ms vs. Tool Unprimed = 1,017 ± 43 ms; Animal Primed = 901 ± 38 ms vs. Animal Unprimed = 962 ± 37 ms). Regarding fMRI, we found that the lexical decision (i.e., words > pseudowords) recruited the left angular, middle frontal (*p* < 0.05 FWE cluster corrected, *p* < 0.001 cluster-forming threshold), and middle temporal gyri (*p* < 0.001 uncorrected; Figure S1A and Table S2A). The general semantic priming effect (i.e., unprimed nouns > primed nouns), irrespective of word semantic category, elicited significant activity in a cluster of the left pars triangularis of the IFG (*p* < 0.05 FWE cluster corrected, *p* < 0.001 cluster-forming threshold; Figure 1E top and Table S2B). The reverse contrast testing for repetition enhancement revealed a left precuneus activation (*p* < 0.05 FWE cluster corrected, *p* < 0.001 cluster-forming threshold; Figure S1B and Table S2C). Regardless of the priming condition, tool nouns, as compared to animal nouns, activated the pars triangularis of the IFG bilaterally, the right superior frontal gyrus extending to the left hemisphere as well (*p* < 0.05 FWE cluster corrected, *p* < 0.001 cluster forming threshold), and the left OTC (*p* < 0.001 uncorrected; Figure 1E bottom and Table S2D). No significant cluster emerged from the opposite contrast meant to identify activations specifically induced by processing animal nouns.

### Semantic Categories Are Decoded Within the Tool-Use Planning Network

**Figure 2.**
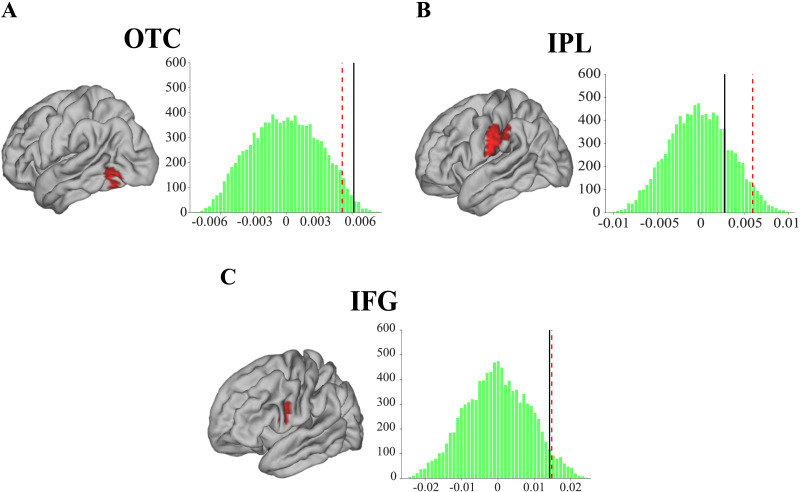
Significant similarity of neural patterns within semantic categories in the left occipito-temporal cortex (OTC) activated by tool use. (A) Significant semantic decoding in the left OTC (mean similarity difference = 0.006 ± 0.003; uncorrected *p* = 0.02; FDR-corrected *p* = 0.04) of the tool-use-planning network. (B) No significant semantic decoding in the left IPL (mean similarity difference = 0.003 ± 0.004; uncorrected *p* = 0.23; FDR-corrected *p* = 0.23). (C) No significant semantic decoding in the left IFG (mean similarity difference = 0.014 ± 0.009; uncorrected *p* = 0.06; FDR-corrected *p* = 0.09). The green histograms represent the distribution obtained after 10,000 permutations of neural-pattern-similarity difference scores. The black line represents the observed group average, and the red dashed line is the unilateral probability threshold set at *p* = 0.05.

To test our prediction that activity within the tool-use network represents tool-related semantic information, we conducted a multivariate analysis on the semantic activation patterns in the left IFG, left IPL, and left OTC, three crucial nodes of the tool-use planning network we identified with the motor task and with a documented role in semantic processing of tool nouns as described earlier (Aflalo et al., 2020; Cattaneo et al., 2010; Chao et al., 1999). We reasoned that if semantic information is encoded within tool-use-related regions, the spatial organization of semantic neural responses elicited within these regions should be similar for tool nouns, whether they are primed or not. The same rationale should apply for animal nouns. The opposite is expected when comparing representations of distinct semantic categories: tool nouns and animal nouns should elicit significantly dissimilar patterns of neural activities in tool-use related regions (contrast 1 in Materials and Methods; see Figure S2 for the similarity matrices of each ROI). In other words, we expected the similarity of neural activity to be higher for words within a semantic category than across categories. To assess this, we measured the distance between the neural patterns for each pair of conditions (i.e., 4 priming conditions resulting in 6 possible pairs, see Materials and Methods). Crucially, the difference in neural similarity between within- and across-semantic-category words was significant in the left OTC, part of the tool-use planning neural network (mean similarity difference = 0.006 ± 0.003; uncorrected *p* = 0.02; FDR-corrected *p* = 0.04; Figure 2A). More precisely, neural patterns in this region were similar for tool nouns on the one hand, and for animal nouns on the other hand, irrespective of priming. This was not the case for the activation patterns recorded in the left IPL (mean similarity difference = 0.003 ± 0.004; uncorrected *p* = 0.23; FDR-corrected *p* = 0.23; Figure 2B). We also performed the same exploratory analysis in the left IFG cluster activated by tool use (see Materials and Methods). The patterns for tool and animal nouns were not successfully decoded in this region (mean similarity difference = 0.014 ± 0.009; uncorrected *p* = 0.06; FDR-corrected *p* = 0.09; Figure 2C). We ran two further contrasts (2 and 3), testing whether one within-category pair could be uniquely represented compared to any other pair (i.e., the other within-category pair and the four across-category pairs). For the tool-nouns pair, we did not find a stronger similarity in the OTC nor in the IPL (uncorrected *p*_s_ > 0.31; FDR-corrected *p*_s_ > 0.31). However, the exploratory analysis in the IFG revealed that tool nouns were specifically represented in this region, as they elicited a stronger within-category similarity than the animal-nouns pair and the four across-category pairs (mean similarity difference = 0.035 ± 0.015; uncorrected *p* = 0.02; FDR-corrected *p* = 0.04; Figure S3). By contrast, the analysis testing for a unique representation of animal nouns did not reveal any significance in any of the ROIs (uncorrected *p*_s_ > 0.06; FDR-corrected *p*_s_ > 0.13). Hence, in the left OTC activated by tool-use planning, the semantic neural activity elicited when processing tool nouns or animal nouns was dissimilar from the one elicited by word pairs belonging to the two different semantic categories. This suggests that neural activity within this OTC contains tool-related, but also animal-related, semantic information. Besides, our exploratory analysis in the left IFG activated by tool-use planning revealed that tool nouns may be uniquely represented in this region given the observed stronger within-category similarity.

### Unprimed Tool Nouns Are the Most Activated Within the OTC

The multivariate analysis run on the OTC demonstrated that both animal and tool categories can be represented within this area. To test whether the tool-use-planning network is recruited specifically by tool nouns in this region, we conducted a follow-up analysis (see Materials and Methods), where we predicted that tool nouns would elicit a stronger signal magnitude than animal nouns. By testing if the averaged neural activity within the left OTC differed across semantic conditions, we found an interaction between Priming and Semantic Category (*F*_(1, 19)_ = 13.6; *p* = 0.002; neural signal for Tool Unprimed = 1.82 ± 0.41; Tool Primed = 1.54 ± 0.44; Animal Unprimed = 1.49 ± 0.37; Animal Primed = 1.59 ± 0.40). Post hoc tests revealed that OTC activity was significantly higher for unprimed tool nouns than for primed tool nouns (*p* = 0.005) and unprimed animal nouns (*p* = 0.02).

## DISCUSSION

The present study aimed to test whether tool-use related neural activity contributes to semantic processing of tool nouns and can dissociate their neural representations from those of animal nouns. Our findings unveil that neural activity within the left occipito-temporal area of the tool-use planning network takes part in semantic decoding of tool nouns. Tool nouns and animal nouns involve distinct representations, and tool nouns evoke higher neural activity than animal nouns in this region. Furthermore, exploratory investigation on the relatively small IFG cluster activated by tool-use planning revealed semantic decoding specific to tool nouns, that is, no within-category similarity for animal nouns nor any across-category similarities in this area. So far, the available neuroimaging evidence was limited to the presupposed co-localization of semantic and motor processes. Critically, however, these studies did not assess the sensorimotor function with actual movements (Boronat et al., 2005; Chao et al., 1999; Chao & Martin, 2000), not to mention tool use, known to recruit a larger neural network than free-hand movements (Brandi et al., 2014; Peeters et al., 2009; Thibault et al., 2021). We went a step further by identifying the clusters activated by tool-use actions and by a semantic task including tool and animal nouns in those very same participants. The recorded neural activity highlighted distinct neural representations for each semantic category within the left occipito-temporal cluster involved in tool-use planning. Both primed and unprimed nouns pertaining to the same semantic category elicited similar neural patterns in this region, while the neural patterns elicited by the two semantic categories (tool nouns and animal nouns) were distinguishable. Furthermore, and crucially, in the left occipito-temporal cluster, tool nouns induced higher neural activity than animal nouns, suggesting that tool-noun representations are strongly established within this tool-use area. This provides compelling evidence that tool-related semantic information is supported by neural resources that also underpin actual tool use. Such findings speak in favor of language embodiment theories (Allport, 1985; Barsalou, 1999, 2008; Barsalou et al., 2003; Borghi & Barsalou, 2021; Buccino et al., 2016; García & Ibáñez, 2016; Pulvermüller, 2005; Pulvermüller & Fadiga, 2010), suggesting that linguistic components such as semantics are embodied within modality-specific systems for action and perception. Indeed, parts of the tool-use sensorimotor network are engaged when accessing semantic information about tool-related words, enabling the decoding of these words, as distinguished from those referring to animals. This concurs with the general idea that semantic representations are supported by modality-specific representations built on sensorimotor experiences (Fernandino et al., 2022). The specific decoding of tool nouns we observed in the left IFG also comes in support of such interpretation. However, the exploratory nature of this analysis invites us to moderate our interpretation, and further studies are needed to better characterize the contribution of the IFG in both tool use and semantic processing.

In the motor domain, neuroimaging studies previously reported a contribution of the left OTC to tool use (Brandi et al., 2014; Peeters et al., 2009). More generally, this cortical area lies at the intersection of the ventral and dorsal streams, coding for object conceptual representations and goal-directed movements of the upper limbs, respectively (Goodale & Milner, 1992; Ungerleider & Mishkin, 1982). This region has also been recently proposed to represent more abstract motor representations (Wurm & Caramazza, 2022). The OTC would encode conceptual information of action that does not depend on the movement kinematics, the context or the manipulated object (e.g., hammering is a back-and-forth movement regardless of the direction of the object involved; Wurm & Caramazza, 2022; Wurm & Lingnau, 2015). The neural patterns for action observation in this area are indeed similar to those elicited when reading a sentence describing the same action, thus revealing the crucial role of the OTC in holding an action concept across different domains (Wurm & Caramazza, 2019). Along this line, our study suggests that abstract representations of a tool-use action may support semantic processing of tool nouns. We did not find evidence for word semantic processing in the left IPL cluster activated by tool use, contrary to the assumptions made by previous work (Boronat et al., 2005; Chao & Martin, 2000). This suggests that semantic processing of object categories within the IPL might occur outside of the tool-use network. The IPL is part of the dorsal stream and plays a role in retrieving knowledge about how to use a tool (Buxbaum & Kalénine, 2020; Lesourd et al., 2021). This knowledge may be less activated in a semantic priming task such as the one we employed here, which may involve more associative knowledge (i.e., a hammer is used to sink a nail; Kalénine & Buxbaum, 2016; Lesourd et al., 2021).

Our finding of common neurofunctional resources between tool-related semantic information and tool use predicts potential reciprocal impact of one function over the other at the behavioral level (Dahlin et al., 2008; Thibault et al., 2021). Coherently, the use of novel tools that received a label, which may have sharpened their semantic representation, is facilitated in comparison with unlabeled tools (Foerster et al., 2020). We also predict the so far untested reverse influence, namely that tool use may benefit semantic processing, in line with the reciprocal impact more generally documented between action and action-related language (Boulenger et al., 2006; Marangolo et al., 2010).

Our work opens further perspectives about the strength of the tool-noun representations within the network recruited for actual tool use. First, it would be of interest to extend the investigations to various types of tools. Even if different tools have been shown to recruit the same brain regions when used (Chen et al., 2023), subtle activation differences may exist and be differently related to the processing of tool nouns. Second, in keeping with the previous point, it may be important to consider whether the tool-use planning network encodes broad semantic information such as object manipulability or, alternatively, encodes semantic aspects that are more specific to the tools themselves. Indeed, objects such as a candle or an apple are manipulable, but they do not act on the physical world in the same way a tool does. Whether semantic representations of manipulable, non-tool objects can be also represented in the proper tool-use network remains to be tested. A recent study revealed behavioral and electrophysiological differences for the processing of nouns and pictures of tool and non-tool objects, suggesting the existence of distinct representations in the brain (Visani, Garofalo, et al., 2022).

Finally, whether these results would generalize to different tasks involving tools, such as observing tool-use actions or imitating and/or imagining them, is an open question. As stated in the Introduction, recent evidence that focused on manual actions (but not tool-use actions) reveals that the functional network for action observation is more similar to action-related language than the network underlying action execution (Courson & Tremblay, 2020). Along this line, it would be interesting to test whether tool-noun representations are more tightly linked to the observation of tool-use actions than to their actual execution. Some lines of evidence showing common brain activity, in the OTC and IFG, for language and the observation of symbolic gestures of both tool-use and non-tool-use actions (Andric et al., 2013; Xu et al., 2009), suggest possible links that need further investigation.

## ACKNOWLEDGMENTS

We thank Raphaël Py and Mattia Gervasi for their support in data acquisition and analyses; François Lecomte for drawing figures of the fMRI experimental setup; Franck Lamberton and Danielle Ibarrola for their support with the MRI facilities; and Livio Finos for recommending statistical analyses.

## FUNDING INFORMATION

Claudio Brozzoli, Swedish Research Council (https://dx.doi.org/10.13039/501100004359), Award ID: 2015-01717. Claudio Brozzoli, Agence Nationale de la Recherche (https://dx.doi.org/10.13039/501100001665), Award ID: ANR-JC (ANR-16-CE28-0008-01). Alice C. Roy, Agence Nationale de la Recherche (https://dx.doi.org/10.13039/501100001665), Award ID: ANR-16-CE28-0015. Alice C. Roy, LabEx ASLAN (https://dx.doi.org/10.13039/501100011602), Award ID: ANR-10-LABX-0081. Alice C. Roy, LabEx ASLAN (https://dx.doi.org/10.13039/501100011602), Award ID: ANR-11-IDEX-0007. Véronique Boulenger, LabEx ASLAN (https://dx.doi.org/10.13039/501100011602), Award ID: ANR-10-LABX-0081. Véronique Boulenger, LabEx ASLAN (https://dx.doi.org/10.13039/501100011602), Award ID: ANR-11-IDEX-0007.

## AUTHOR CONTRIBUTIONS

**Simon Thibault**: Conceptualization; Data curation; Formal analysis; Investigation; Methodology; Software; Validation; Visualization; Writing – original draft; Writing – review & editing. **Eric Koun**: Methodology; Resources; Software. **Romeo Salemme**: Methodology; Resources; Software. **Alice C. Roy**: Conceptualization; Funding acquisition; Methodology; Project administration; Resources; Supervision; Validation; Writing – review & editing. **Véronique Boulenger**: Conceptualization; Funding acquisition; Methodology; Project administration; Resources; Supervision; Validation; Writing – review & editing. **Claudio Brozzoli**: Conceptualization; Funding acquisition; Methodology; Project administration; Resources; Supervision; Validation; Writing – review & editing.

## DATA AND CODE AVAILABILITY STATEMENT

Data and codes are available at https://osf.io/r9hmd/.

## Supplementary Material


