# Supplementary material for: Activity in Occipito-Temporal Cortex Is Involved in Tool-Use Planning and Contributes to Tool-Related Semantic Neural Representations
Source: Neurobiol Lang (Camb). 2024 Dec 3;5(4):1008–24. doi: 10.1162/nol_a_00159 (PMC11620707; doi:10.1162/nol_a_00159)
Supplement: Supplementary file 1 [file nol-5-4-1008-s001.pdf]

Supplementary Information

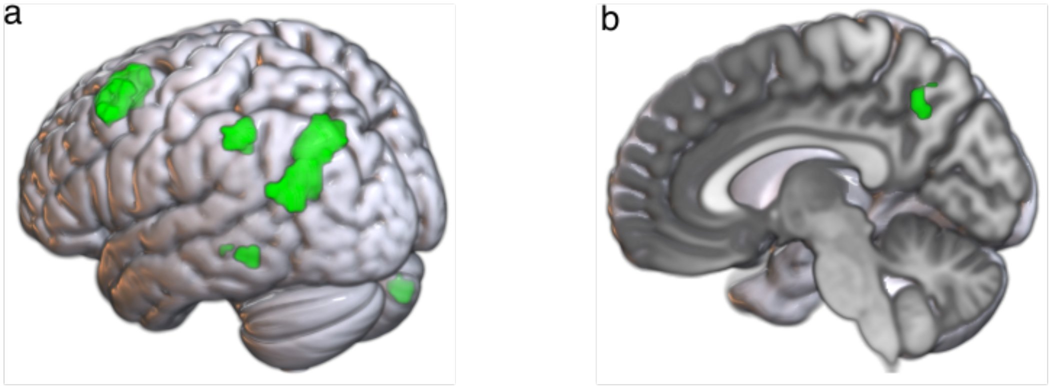

Figure S1. Brain activations for the lexical decision task derived from the contrast *words – pseudowords* (a) and the main effect of repetition enhancement derived from the contrast *primed nouns – unprimed nouns* (b), uncorrected maps at $p < 0.001$ (see Table S2 for clusters passing the FWE correction at the cluster level).

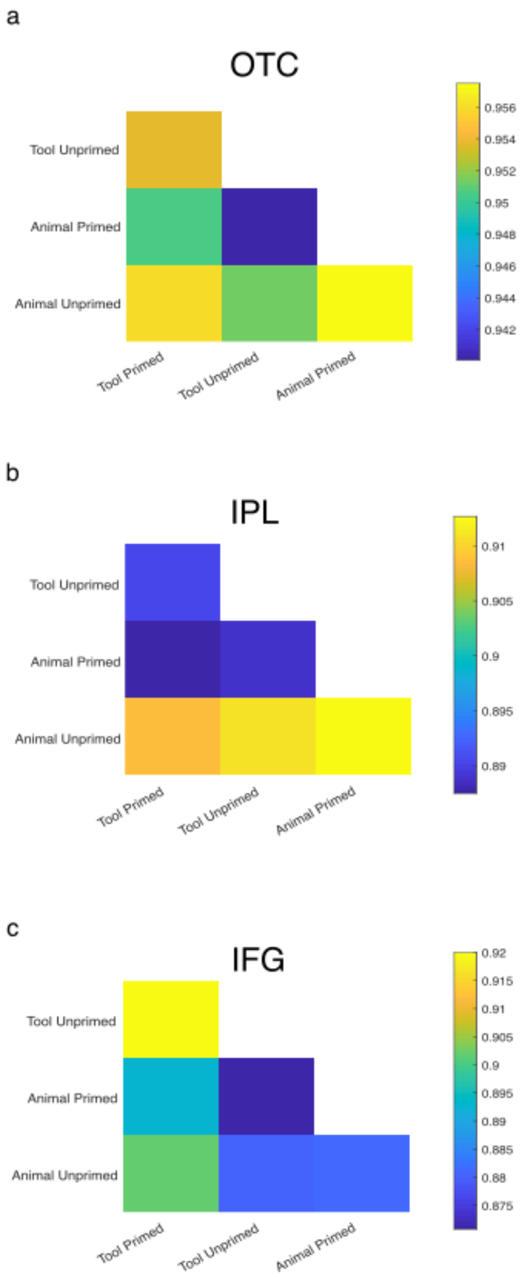

Figure S2: Figure S2: Neural similarity matrices for OTC (a), IPL (b) and IFG (c). Yellow colors represent greater similarities and blue colors lower similarities between conditions. The scales on the right represent the measure of similarity.

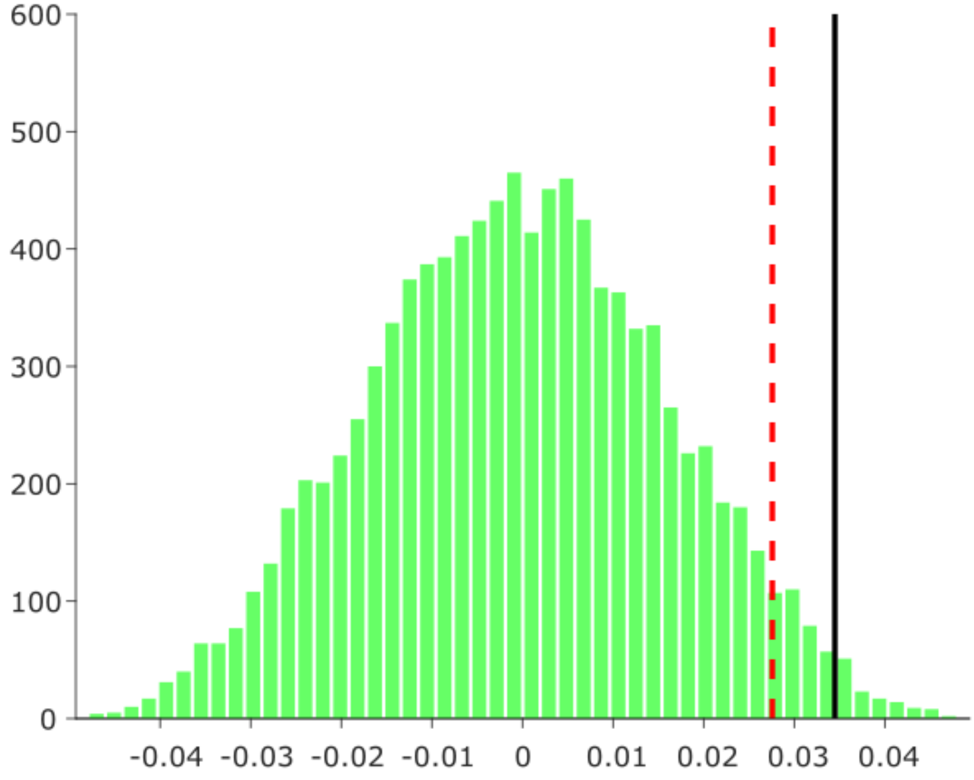

Figure S3: Significant tool-nouns semantic decoding in the left IFG (mean similarity difference = 0.035 ± 0.015; uncorrected p = 0.02; FDR-corrected p = 0.04) of the tool-use-planning network. The green histograms represent the distribution obtained after 10,000 permutations of neural patterns similarity difference scores. The black line represents the observed group average, and the red dashed line is the unilateral probability threshold set at p=0.05.

Region	BA	Peak MNI coordinates			Cluster size (*k*)	Z-value
		x	*y*	*z*		
**(A) Tool-use Planning Network: (Tool-use $_{Planning}$ – Free-Hand $_{Planning}$) - (Tool-use $_{Execution}$ – Free-Hand $_{Execution}$)**						
**L Postcentral Gyrus (extending onto Inferior Parietal Lobule)[FWE]**	**BA1, 2, 3, 40**	**-66**	**-16**	**27**	**150**	**4.64**
L Caudate (extending onto Globus Pallidus)[FWE]	-	-18	20	-13	112	4.31
**L Occipitotemporal Cortex[FWE]**	**BA19, 37**	**-54**	**-72**	**-9**	**76**	**4.53**
L Putamen	-	-28	-4	-6	46	4.03
**L Inferior Frontal Gyrus (Precentral Suclus)**	**BA6**	**-58**	**12**	**27**	**28**	**3.65**
L Intraparietal Sulcus	BA40	-36	-36	44	26	4.18
L Putamen	-	-24	8	-16	21	4.34
L Insula	BA13	-42	-4	4	19	3.74
L Ventral Anterior Cingulate Cortex	BA24	-10	30	11	17	4.40
L Inferior Frontal Gyrus (Ventral Premotor Cortex)	BA6	-64	0	30	11	4.15

R Putamen (cluster extending onto Globus Pallidus and Caudate Nucleus) FWE	-	26	-18	-3	772	5.21
R Inferior Frontal Gyrus (extending onto Postcentral and Supramarginal Gyri)FWE	BA1,2,3,4,6,40	62	6	30	177	4.37
R Occipitotemporal CortexFWE	BA19, 37	50	-58	-9	141	4.72
R Intraparietal Sulcus	BA7	36	-42	47	130	4.55
R Secondary Visual Area	BA18	26	-100	-6	13	3.65
R Cerebellum Crus I	-	26	-46	-39	11	3.65
**(B) Tool-use Execution Network: (Tool-use $_{Planning}$ – Free-Hand $_{Planning}$) - (Tool-use $_{Execution}$ – Free-Hand $_{Execution}$)**						
L Secondary Visual Area (extending bilaterally)FWE	BA18	-12	-78	-3	590	7.27

Table S1. Brain areas activated for tool-use planning (A) and tool-use execution (B). All presented clusters contain more than 10 contiguous voxels and are below the statistics threshold $p < 0.001$, unc. Clusters passing the family-wise error correction with $p < 0.05$ at the cluster level are indicated with the mention FWE. The three regions highlighted are the ROIs used for the multivariate analyses focusing on the neural activity elicited by the semantic priming task.

Region	BA	Peak MNI coordinates			Cluster size (*k*)	Z-value
		x	*y*	*z*		
**(A) Words - Pseudowords**						
L Inferior Angular Gyrus[FWE]	BA39	-34	-78	47	236	4.71
L Middle Frontal Gyrus[FWE]	BA6	-42	12	60	186	4.51
L Superior Angular Gyrus[FWE]	BA39	-60	-48	47	46	4.12
L Middle Temporal Gyrus	BA21	-60	-54	-6	26	4.43
R Cerebellum Crus II	-	38	-76	-46	21	4.22
**(B) Unprimed nouns - Primed nouns**						
L Inferior Frontal Gyrus (*Pars Triangularis*)[FWE]	BA45	-30	30	4	54	4.32
L Inferior Frontal Gyrus (*Pars Opercularis*)	BA44	-48	20	14	26	4.02
L Superior Frontal Gyrus	BA8	-6	20	53	17	3.43
L Superior Frontal Gyrus (Supplementary Motor Area)	BA6	-10	12	57	11	3.47
**(C) Primed nouns - Unprimed nouns**						
L Precuneus[FWE]	BA7	-6	-52	53	49	4.02
LCaudate	-	-10	26	9	12	3.71
L Superior Frontal Gyrus	BA6	-16	-16	57	11	3.80
R Superior Temporal Gyrus	BA22	54	2	-6	16	3.79
R Fusiform Gyrus	BA37	30	-52	-13	11	3.91
**(D) Tool nouns - Animal nouns**						
L Inferior Frontal Gyrus (*Pars Orbitalis*)[FWE]	BA47	-42	42	1	49	3.87
L Anterior Insula	BA13	-34	20	-3	34	4.59
L Occipitotemporal Cortex	BA37	-48	-52	-13	18	4.13

R Superior Frontal Gyrus (Supplementary Motor Area, extending bilaterally)^{FWE}	BA6	8	14	50	88	4.67
R Inferior Frontal Gyrus (*Pars Orbitalis*)^{FWE}	BA47	36	24	-6	44	4.86
R Middle Frontal Gyrus	BA9	42	30	24	17	3.53
R Caudate Nucleus	-	2	0	17	11	3.86

Table S2. Brain areas activated for the lexical decision effect (A), the main effect of semantic priming irrespective of word categories (B), the main effect of repetition enhancement (C) and the main effect of the tool semantic category (D). All reported clusters contain more than 10 contiguous voxels and are below the statistics threshold $p < 0.001$, unc. Clusters passing the family-wise error correction with $p < 0.05$ at the cluster level are indicated with the mention ^{FWE}. No significant voxel was found for the main effect of the animal semantic category (i.e. Animal nouns – Tool nouns).

	Tool Nouns	Animal Nouns	Semantic Categories Statistics	Word Lists Statistics
Written Frequency	7.27 ± 8.98	6.82 ± 6.23	W=2720.5, p=0.26	$F_{(9,130)}$=1.18, p =0.31
Oral Frequency	4.65 ± 7.42	5.45 ± 6.53	$t_{(138)}$=1.57, p =0.12	$F_{(9,130)}$=1.12, p =0.35
Letters	6.76 ± 1.86	6.26 ± 1.98	W=2109.5, p=0.15	$F_{(9,130)}$=0.87, p =0.56
Syllables	1.96 ± 0.81	1.86 ± 0.82	W=2277.5, p=0.44	$\chi^2_{(9)}$=9.79, p=0.37
Orthographic Neighbors	3.40 ± 4.21	2.60 ± 4.02	W=2045, p=0.08	$\chi^2_{(9)}$=8.91, p=0.45
Unknown Words	0.59 ± 0.92	0.34 ± 0.74	W=2080, p=0.06	$\chi^2_{(9)}$=12.83, p=0.17
Imageability	6.59 ± 0.30	6.44 ± 0.60	W=2313.5, p=0.57	$\chi^2_{(9)}$=9.80, p=0.37
Manipulability	5.90 ± 0.35	×	×	$F_{(4,65)}$=0.69, p =0.60

Table S3. Statistics for tool and animal nouns. Means ± SD are reported respectively for tool and animal nouns on several psycholinguistic variables: written and oral frequencies (occurrences per million), number of letters, number of syllables, number of orthographic neighbors, the times a word is reported unknown, imageability and, for tool nouns, manipulability. Statistics are reported for the comparison between the two semantic categories (i.e. 2 levels, animals vs. tools; two-sample t-test or Wilcoxon sum rank test) as well as between the 10 lists of words used either as primes or targets across the five experimental conditions (i.e. 10 levels; ANOVA or Kruskal-Wallis test performed according to the residuals normality).

Prime	Target	Prime (translation)	Target (translation)	Condition
pieuvre	dauphin	octopus	dolphin	Animal Primed
cafard	araignée	cockroach	spider	Animal Primed
boeuf	vache	beef	cow	Animal Primed
chèvre	bouc	goat	goat	Animal Primed
pie	mouette	magpie	seagull	Animal Primed
crabe	oursin	crab	sea urchin	Animal Primed
libellule	papillon	dragonfly	butterfly	Animal Primed
lion	panthère	lion	panther	Animal Primed
cerf	biche	stag	doe	Animal Primed
rhinocéros	zèbre	rhinoceros	zebra	Animal Primed
corbeau	oie	crow	goose	Animal Primed
paon	autruche	peacock	ostrich	Animal Primed
guêpe	abeille	wasp	bee	Animal Primed
grenouille	vipère	frog	viper	Animal Primed
craie	stylo	chalk	pen	Tool Primed
javelot	raquette	javelin	racket	Tool Primed
brosse	peigne	brush	comb	Tool Primed
fouet	lasso	whip	lasso	Tool Primed
aspirateur	balai	vacuum	broom	Tool Primed
gomme	crayon	rubber	pencil	Tool Primed
équerre	compas	square	compass	Tool Primed
feutre	pinceau	felt	brush	Tool Primed
seringue	pipette	syringe	pipette	Tool Primed
décapsuleur	louche	bottle opener	ladle	Tool Primed
rame	pagaie	oar	paddle	Tool Primed
hache	machette	chopped	machete	Tool Primed
tronçonneuse	scie	chain saw	saw	Tool Primed
briquet	allumette	lighter	matche	Tool Primed
pelle	brebis	shovel	ewe	Animal Unprimed
tournevis	fourmi	screwdriver	ant	Animal Unprimed
épée	girafe	sword	giraffe	Animal Unprimed
lime	panda	lime	panda	Animal Unprimed
scalpel	puma	scalpel	puma	Animal Unprimed
pioche	koala	pickaxe	koala	Animal Unprimed
poignard	brochet	dagger	pike	Animal Unprimed

pince	écrevisse	pliers	crayfish	Animal Unprimed
hachoir	mésange	chopper	tit	Animal Unprimed
tamis	hippopotame	sieve	hippopotamus	Animal Unprimed
fourche	gorille	fork	gorilla	Animal Unprimed
aiguille	rat	needle	rat	Animal Unprimed
balayette	coq	brush	rooster	Animal Unprimed
crosse	loup	butt	wolf	Animal Unprimed
tortue	manivelle	turtle	crank	Tool Unprimed
mouche	agrafeuse	fly	stapler	Tool Unprimed
taureau	spatule	taurus	spatula	Tool Unprimed
cygne	stylet	swan	stylus	Tool Unprimed
mouton	cisaille	sheep	shears	Tool Unprimed
hippocampe	rasoir	seahorse	razoe	Tool Unprimed
coccinelle	batte	ladybug	bat	Tool Unprimed
lynx	chalumeau	lynx	blowtorch	Tool Unprimed
daim	arrosoir	deer	watering can	Tool Unprimed
âne	éplucheur	donkey	peeler	Tool Unprimed
canard	télécommande	duck	remote control	Tool Unprimed
perroquet	clef	parrot	key	Tool Unprimed
bourdon	canne	bumblebee	cane	Tool Unprimed
lézard	ponceuse	lizard	sander	Tool Unprimed
sanglier	tupou	boar	tupou	Pseudowords
aigle	telcite	eagle	telcite	Pseudowords
escargot	crapal	snail	crapal	Pseudowords
guenon	celnar	monkey	celnar	Pseudowords
hérisson	soricame	hedgehog	soricame	Pseudowords
écureuil	paceton	squirrel	paceton	Pseudowords
renard	létupien	fox	létupien	Pseudowords
grillon	fulet	cricket	fulet	Pseudowords
veau	gralut	calf	gralut	Pseudowords
crevette	permoriteur	shrimp	permoriteur	Pseudowords
huître	faurmeau	oyster	faurmeau	Pseudowords
ours	paindon	bear	paindon	Pseudowords
limace	granpoir	slug	granpoir	Pseudowords
salamandre	greplin	salamander	greplin	Pseudowords
râteau	cruine	rake	cruine	Pseudowords

marteau	ladontin	hammer	ladontin	Pseudowords
sabre	majot	saber	majot	Pseudowords
râpe	tranon	grated	tranon	Pseudowords
cutter	panton	cutter	panton	Pseudowords
massue	carporan	club	carporan	Pseudowords
canif	bimette	penknife	bimette	Pseudowords
sécateur	solaie	shears	solaie	Pseudowords
couteau	orunel	knife	orunel	Pseudowords
épuisette	rinot	net	rinot	Pseudowords
truelle	bafin	trowel	bafin	Pseudowords
perceuse	daillot	drill	daillot	Pseudowords
soufflet	pimeau	bellows	pimeau	Pseudowords
levier	riploir	lever	riploir	Pseudowords

Table S4. List of Prime-Target pairs of stimuli in French with their English translation and the corresponding conditions for the semantic task.

fMRIprep citation file

Results included in this manuscript come from preprocessing performed using *fMRIPrep* 20.2.0 (Esteban, Markiewicz, et al. (2018); Esteban, Blair, et al. (2018); RRID:SCR_016216), which is based on *Nipype* 1.5.1 (Gorgolewski et al. (2011); Gorgolewski et al. (2018); RRID:SCR_002502).

Anatomical data preprocessing

A total of 2 T1-weighted (T1w) images were found within the input BIDS dataset. All of them were corrected for intensity non-uniformity (INU) with N4BiasFieldCorrection (Tustison et al. 2010), distributed with ANTs 2.3.3 (Avants et al. 2008, RRID:SCR_004757). The T1w-reference was then skull-stripped with a *Nipype* implementation of the antsBrainExtraction.sh workflow (from ANTs), using OASIS30ANTs as target template. Brain tissue segmentation of cerebrospinal fluid (CSF), white-matter (WM) and gray-matter (GM) was performed on the brain-extracted T1w using fast (FSL 5.0.9, RRID:SCR_002823, Zhang, Brady, and Smith 2001). A T1w-reference map was computed after registration of 2 T1w images (after INU-correction) using mri_robust_template (FreeSurfer 6.0.1, Reuter, Rosas, and Fischl 2010). Brain surfaces were reconstructed using recon-all (FreeSurfer 6.0.1, RRID:SCR_001847, Dale, Fischl, and Sereno 1999), and the brain mask estimated previously was refined with a custom variation of the method to reconcile ANTs-derived and FreeSurfer-derived segmentations of the cortical gray-matter of Mindboggle (RRID:SCR_002438, Klein et al. 2017). Volume-based spatial normalization to one standard space (MNI152NLin2009cAsym) was performed through nonlinear registration with antsRegistration (ANTs 2.3.3), using brain-extracted versions of both T1w reference and the T1w template. The following template was selected for spatial normalization: *ICBM 152 Nonlinear Asymmetrical template version 2009c* [Fonov et al. (2009), RRID:SCR_008796; TemplateFlow ID: MNI152NLin2009cAsym],

Functional data preprocessing

For each of the 11 BOLD runs found per subject (across all tasks and sessions), the following preprocessing was performed. First, a reference volume and its skull-stripped version were generated using a custom methodology of *fMRIPrep*. A B0-nonuniformity map (or *fieldmap*) was estimated based on a phase-difference map calculated with a dual-echo GRE (gradient-recall echo) sequence, processed with a custom workflow of *SDCFlows* inspired by the epidewarp.fsl script and further improvements in HCP Pipelines (Glasser et al. 2013). The *fieldmap* was then co-registered to the target EPI (echo-planar imaging) reference run and converted to a displacements field map (amenable to registration tools such as ANTs) with FSL's fugue and other *SDCflows* tools. Based on the estimated susceptibility distortion, a corrected EPI (echo-planar imaging) reference was calculated for a more accurate co-registration with the anatomical reference. The BOLD reference was then co-registered to the T1w reference using bbregister (FreeSurfer) which implements boundary-based registration (Greve and Fischl 2009). Co-registration was

configured with six degrees of freedom. Head-motion parameters with respect to the BOLD reference (transformation matrices, and six corresponding rotation and translation parameters) are estimated before any spatiotemporal filtering using mcflirt (FSL 5.0.9, Jenkinson et al. 2002). BOLD runs were slice-time corrected using 3dTshift from AFNI 20160207 (Cox and Hyde 1997, RRID:SCR_005927). The BOLD time-series (including slice-timing correction when applied) were resampled onto their original, native space by applying a single, composite transform to correct for head-motion and susceptibility distortions. These resampled BOLD time-series will be referred to as *preprocessed BOLD in original space*, or just *preprocessed BOLD*. The BOLD time-series were resampled into standard space, generating a *preprocessed BOLD run in MNI152NLin2009cAsym space*. First, a reference volume and its skull-stripped version were generated using a custom methodology of *fMRIPrep*. Several confounding time-series were calculated based on the *preprocessed BOLD*: framewise displacement (FD), DVARS and three region-wise global signals. FD was computed using two formulations following Power (absolute sum of relative motions, Power et al. (2014)) and Jenkinson (relative root mean square displacement between affines, Jenkinson et al. (2002)). FD and DVARS are calculated for each functional run, both using their implementations in *Nipype* (following the definitions by Power et al. 2014). The three global signals are extracted within the CSF, the WM, and the whole-brain masks. Additionally, a set of physiological regressors were extracted to allow for component-based noise correction (*CompCor*, Behzadi et al. 2007). Principal components are estimated after high-pass filtering the *preprocessed BOLD* time-series (using a discrete cosine filter with 128s cut-off) for the two *CompCor* variants: temporal (tCompCor) and anatomical (aCompCor). tCompCor components are then calculated from the top 2% variable voxels within the brain mask. For aCompCor, three probabilistic masks (CSF, WM and combined CSF+WM) are generated in anatomical space. The implementation differs from that of Behzadi et al. in that instead of eroding the masks by 2 pixels on BOLD space, the aCompCor masks are subtracted a mask of pixels that likely contain a volume fraction of GM. This mask is obtained by dilating a GM mask extracted from the FreeSurfer's *aseg* segmentation, and it ensures components are not extracted from voxels containing a minimal fraction of GM. Finally, these masks are resampled into BOLD space and binarized by thresholding at 0.99 (as in the original implementation). Components are also calculated separately within the WM and CSF masks. For each CompCor decomposition, the *k* components with the largest singular values are retained, such that the retained components' time series are sufficient to explain 50 percent of variance across the nuisance mask (CSF, WM, combined, or temporal). The remaining components are dropped from consideration. The head-motion estimates calculated in the correction step were also placed within the corresponding confounds file. The confound time series derived from head motion estimates and global signals were expanded with the inclusion of temporal derivatives and quadratic terms for each (Satterthwaite et al. 2013). Frames that exceeded a threshold of 0.5 mm FD or 1.5 standardised DVARS were annotated as motion outliers. All resamplings can be performed with *a single interpolation step* by composing all the pertinent transformations (i.e. head-motion transform matrices, susceptibility distortion correction when available, and co-registrations to anatomical and output spaces). Gridded (volumetric) resamplings were performed using antsApplyTransforms (ANTs), configured with Lanczos interpolation to minimize

the smoothing effects of other kernels (Lanczos 1964). Non-gridded (surface) resamplings were performed using mri_vol2surf (FreeSurfer).

Many internal operations of *fMRIPrep* use *Nilearn* 0.6.2 (Abraham et al. 2014, RRID:SCR_001362), mostly within the functional processing workflow. For more details of the pipeline, see the section corresponding to workflows in *fMRIPrep*'s documentation.

Copyright Waiver

The above boilerplate text was automatically generated by fMRIPrep with the express intention that users should copy and paste this text into their manuscripts *unchanged*. It is released under the CC0 license.

References

Abraham, Alexandre, Fabian Pedregosa, Michael Eickenberg, Philippe Gervais, Andreas Mueller, Jean Kossaifi, Alexandre Gramfort, Bertrand Thirion, and Gael Varoquaux. 2014. "Machine Learning for Neuroimaging with Scikit-Learn." *Frontiers in Neuroinformatics* 8. https://doi.org/10.3389/fninf.2014.00014.

Avants, B.B., C.L. Epstein, M. Grossman, and J.C. Gee. 2008. "Symmetric Diffeomorphic Image Registration with Cross-Correlation: Evaluating Automated Labeling of Elderly and Neurodegenerative Brain." *Medical Image Analysis* 12 (1): 26–41. https://doi.org/10.1016/j.media.2007.06.004.

Behzadi, Yashar, Khaled Restom, Joy Liau, and Thomas T. Liu. 2007. "A Component Based Noise Correction Method (CompCor) for BOLD and Perfusion Based fMRI." *NeuroImage* 37 (1): 90–101. https://doi.org/10.1016/j.neuroimage.2007.04.042.

Cox, Robert W., and James S. Hyde. 1997. "Software Tools for Analysis and Visualization of fMRI Data." *NMR in Biomedicine* 10 (4-5): 171–78. https://doi.org/10.1002/(SICI)1099-1492(199706/08)10:4/5<171::AID-NBM453>3.0.CO;2-L.

Dale, Anders M., Bruce Fischl, and Martin I. Sereno. 1999. "Cortical Surface-Based Analysis: I. Segmentation and Surface Reconstruction." *NeuroImage* 9 (2): 179–94. https://doi.org/10.1006/nimg.1998.0395.

Esteban, Oscar, Ross Blair, Christopher J. Markiewicz, Shoshana L. Berleant, Craig Moodie, Feilong Ma, Ayse Ilkay Isik, et al. 2018. "FMRIPrep." *Software*. Zenodo. https://doi.org/10.5281/zenodo.852659.

Esteban, Oscar, Christopher Markiewicz, Ross W Blair, Craig Moodie, Ayse Ilkay Isik, Asier Erramuzpe Aliaga, James Kent, et al. 2018. "fMRIPrep: A Robust Preprocessing Pipeline for Functional MRI." *Nature Methods*. https://doi.org/10.1038/s41592-018-0235-4.

Fonov, VS, AC Evans, RC McKinstry, CR Almli, and DL Collins. 2009. “Unbiased Nonlinear Average Age-Appropriate Brain Templates from Birth to Adulthood.” *NeuroImage* 47, Supplement 1: S102. https://doi.org/10.1016/S1053-8119(09)70884-5.

Glasser, Matthew F., Stamatios N. Sotiropoulos, J. Anthony Wilson, Timothy S. Coalson, Bruce Fischl, Jesper L. Andersson, Junqian Xu, et al. 2013. “The Minimal Preprocessing Pipelines for the Human Connectome Project.” *NeuroImage*, Mapping the connectome, 80: 105–24. https://doi.org/10.1016/j.neuroimage.2013.04.127.

Gorgolewski, K., C. D. Burns, C. Madison, D. Clark, Y. O. Halchenko, M. L. Waskom, and S. Ghosh. 2011. “Nipype: A Flexible, Lightweight and Extensible Neuroimaging Data Processing Framework in Python.” *Frontiers in Neuroinformatics* 5: 13. https://doi.org/10.3389/fninf.2011.00013.

Gorgolewski, Krzysztof J., Oscar Esteban, Christopher J. Markiewicz, Erik Ziegler, David Gage Ellis, Michael Philipp Notter, Dorota Jarecka, et al. 2018. “Nipype.” *Software*. Zenodo. https://doi.org/10.5281/zenodo.596855.

Greve, Douglas N, and Bruce Fischl. 2009. “Accurate and Robust Brain Image Alignment Using Boundary-Based Registration.” *NeuroImage* 48 (1): 63–72. https://doi.org/10.1016/j.neuroimage.2009.06.060.

Jenkinson, Mark, Peter Bannister, Michael Brady, and Stephen Smith. 2002. “Improved Optimization for the Robust and Accurate Linear Registration and Motion Correction of Brain Images.” *NeuroImage* 17 (2): 825–41. https://doi.org/10.1006/nimg.2002.1132.

Klein, Arno, Satrajit S. Ghosh, Forrest S. Bao, Joachim Giard, Yrjö Häme, Eliezer Stavsky, Noah Lee, et al. 2017. “Mindboggling Morphometry of Human Brains.” *PLOS Computational Biology* 13 (2): e1005350. https://doi.org/10.1371/journal.pcbi.1005350.

Lanczos, C. 1964. “Evaluation of Noisy Data.” *Journal of the Society for Industrial and Applied Mathematics Series B Numerical Analysis* 1 (1): 76–85. https://doi.org/10.1137/0701007.

Power, Jonathan D., Anish Mitra, Timothy O. Laumann, Abraham Z. Snyder, Bradley L. Schlaggar, and Steven E. Petersen. 2014. “Methods to Detect, Characterize, and Remove Motion Artifact in Resting State fMRI.” *NeuroImage* 84 (Supplement C): 320–41. https://doi.org/10.1016/j.neuroimage.2013.08.048.

Reuter, Martin, Herminia Diana Rosas, and Bruce Fischl. 2010. “Highly Accurate Inverse Consistent Registration: A Robust Approach.” *NeuroImage* 53 (4): 1181–96. https://doi.org/10.1016/j.neuroimage.2010.07.020.

Satterthwaite, Theodore D., Mark A. Elliott, Raphael T. Gerraty, Kosha Ruparel, James Loughead, Monica E. Calkins, Simon B. Eickhoff, et al. 2013. “An improved framework

for confound regression and filtering for control of motion artifact in the preprocessing of resting-state functional connectivity data." *NeuroImage* 64 (1): 240–56. https://doi.org/10.1016/j.neuroimage.2012.08.052.

Tustison, N. J., B. B. Avants, P. A. Cook, Y. Zheng, A. Egan, P. A. Yushkevich, and J. C. Gee. 2010. "N4ITK: Improved N3 Bias Correction." *IEEE Transactions on Medical Imaging* 29 (6): 1310–20. https://doi.org/10.1109/TMI.2010.2046908.

Zhang, Y., M. Brady, and S. Smith. 2001. "Segmentation of Brain MR Images Through a Hidden Markov Random Field Model and the Expectation-Maximization Algorithm." *IEEE Transactions on Medical Imaging* 20 (1): 45–57. https://doi.org/10.1109/42.906424.
